# Hypercholesterolemia Impaired Sperm Functionality in Rabbits

**DOI:** 10.1371/journal.pone.0013457

**Published:** 2010-10-18

**Authors:** Tania E. Saez Lancellotti, Paola V. Boarelli, Maria A. Monclus, Maria E. Cabrillana, Marisa A. Clementi, Leandro S. Espínola, Jose L. Cid Barría, Amanda E. Vincenti, Analia G. Santi, Miguel W. Fornés

**Affiliations:** 1 Laboratorio de Investigaciones Andrológicas de Mendoza (LIAM), Instituto de Histología y Embriología de Mendoza (IHEM), Facultad de Ciencias Médicas, Universidad Nacional de Cuyo – Centro Científico Tecnológico (CCT), Mendoza – Consejo Nacional de Investigaciones Científicas y Técnicas (CONICET), Mendoza, Argentina; 2 Facultad de Ciencias Médicas, Instituto de Investigaciones, Universidad del Aconcagua, Mendoza, Argentina; 3 Laboratorio de Servicios y Ensayos, Instituto Nacional de Tecnología Industrial (INTI)-Frutas y Hortalizas, Luján de Cuyo, Mendoza, Argentina; University of North Dakota, United States of America

## Abstract

Hypercholesterolemia represents a high risk factor for frequent diseases and it has also been associated with poor semen quality that may lead to male infertility. The aim of this study was to analyze semen and sperm function in diet-induced hypercholesterolemic rabbits. Twelve adult White New Zealand male rabbits were fed *ad libitum* a control diet or a diet supplemented with 0.05% cholesterol. Rabbits under cholesterol-enriched diet significantly increased total cholesterol level in the serum. Semen examination revealed a significant reduction in semen volume and sperm motility in hypercholesterolemic rabbits (HCR). Sperm cell morphology was seriously affected, displaying primarily a “folded head”-head fold along the major axe-, and the presence of cytoplasmic droplet on sperm flagellum. Cholesterol was particularly increased in acrosomal region when detected by filipin probe. The rise in cholesterol concentration in sperm cells was determined quantitatively by Gas chromatographic-mass spectrometric analyses. We also found a reduction of protein tyrosine phosphorylation in sperm incubated under capacitating conditions from HCR. Interestingly, the addition of Protein Kinase A pathway activators -dibutyryl-cyclic AMP and iso-butylmethylxanthine- to the medium restored sperm capacitation. Finally, it was also reported a significant decrease in the percentage of reacted sperm in the presence of progesterone. In conclusion, our data showed that diet-induced hypercholesterolemia adversely affects semen quality and sperm motility, capacitation and acrosomal reaction in rabbits; probably due to an increase in cellular cholesterol content that alters membrane related events.

## Introduction

Cholesterol (chol) is a steroid lipid found in the cell membranes and transported in the blood plasma of all animals. However excessive levels of chol in blood circulation (hypercholesterolemia), are strongly associated with progression of atherosclerosis [1]. Chol is an essential component of mammalian plasma membranes (PM) where it is required to establish proper membrane permeability and fluidity [2]. Within the PM, chol has also been implicated in cell signaling processes [3]. Changes in the organization of membrane lipids can have profound consequences on cellular functions such as signal transduction and membrane trafficking [4], [5].

The lipid bilayer of the rabbit sperm membrane, as other mammalian sperm cells, consists mainly of phospholipids and chol, at a molar ratio of 1.5 [6]. Cholesterol is also abundant in other subfractions of rabbit semen (seminal plasma and droplets). Sperm membrane undergoes several modifications from the testis, were they are produced, to the female tract. Membrane lipids, especially chol, are responsible for changes in membrane fluidity and cell responsiveness to the environment, alterations involved in a series of physiological events that are unique for these cells [7]. Chol efflux from PM leads to changes in membrane structure and fluidity that give rise to the sperm capacitated state [8]. Capacitation is defined as the time-dependent acquisition of fertilization competence [9], ability acquired by the sperm during its transit through the female tract. This process involves a PKA-regulated increase in tyrosine phosphorylation (p-Y) of a subset of proteins [4], [10], [11], and is generally assessed as the ability of the acrosome-intact sperm to undergo AR in response to physiological inducers such as the zona pellucida or progesterone [10], [12].

Animals fed with saturated fat-enriched diets raise their plasmatic chol levels and this would have impact on the cell-specific lipid equilibrium between chol and phospholipids that organize the PM [13]. The later modification could affect cellular functions as signal transduction pathways coupled to membrane chol. Sperm membrane lipids are highly responsive to dietary manipulation [13]. Chol-rich diets have been shown to produce a decrease in sperm AR kinetics [14], and detrimental effects on Leydig and Sertoli cell secretory capacity in rabbits [15]. Moreover, previous works showed that human male infertility might be associated with altered lipid metabolism in seminal plasma [16].

The present study aimed at investigating the effects of diet-induced hypercholesterolemia on rabbit semen and sperm physiology, membrane cholesterol concentration, cell motility, capacitation and acrosome reaction.

## Materials and Methods

### Ethics statement

The animal studies described here were reviewed and approved by the animal care and use committees of School of Medicine – National University of Cuyo (Institutional Committee for Use of Laboratory Animals, IACUC).

### Reagents

Unless otherwise stated, all chemicals and solvents of the highest grade available were obtained from Sigma (St. Louis, MO, USA) and Merck (Darmstadt, Germany).

### Animals and diets

For the purposes of this study, twelve fertile male White New Zealand rabbits (1.5 months old of age, acquired from“Don Cipriano” Farm, Mendoza, Argentina) were caged individually for 11 months with a photoperiod of 12 hours light/day and a temperature ranging from 18–25°C. Animals were fed *ad libitum* a standard rabbit diet composed of 17% crude protein, 16% fiber, 2% minimal ether extract (0% saturated fat), 5.3% minerals (data from the manufacturer's analysis, GEPSA FEEDS®). At five months of age, rabbits were split in two groups (6 each) maintaining the average of body weight in both experimental groups (0.937±0.052 kg). A first group, which served as control (designated normal cholesterolemic rabbits, NCR), continued fed with standard cereal-based chow for the specie; and the other group (hypercholesterolemic rabbits, HCR) was fed with experimental diet, ED: 15% crude protein, 14% fiber, 13.5 fat (6.5% saturated fat). The ED was prepared by heating (up to 60°C) 200 g of fat derived from caw named “primer jugo bovino” (Juan Lopez y CIA®; composed by 55% saturated fat). The term “primer jugo bovino” (solidified fat juice) corresponds to specific topic of Argentinian Alimentary Code (www.anmat.gov.ar/codigoa/CAPITULO_VII_Grasos(actualiz11-06).pdf; artículo 543 – (resolución 2012, 19.10.84)). Briefly, this solid corresponds to the cooling of the liquid obtained after subjecting to 80°C adipose tissue from bovine (*Bos taurus*). When this solid was exposed to 60°C it was obtained melted oil. This oil was poured over 1.5 kg of stock diet and thoroughly mechanically mixed. The diet was stored in darkness under refrigeration until used to avoid peroxidation. The resulting stock ED was enriched up to 6.5% saturated fat and 0.05% chol (Instituto Nacional de Tecnología Industrial, INTI). Food intake, body weight (BW), body length (BL) and body mass index (BMI) were recorded weekly. Body length was defined as the distance from the tip of the nose to the anus measured in m and BMI [17] correspond to weight in kg/square of the length expressed in m (BMI  =  BW/BL^2^).

### Plasma lipids

Plasma chol was determined twice monthly from their arrival to our animal facility. Blood samples were collected from the marginal ear vein of non anesthetized animals fasted overnight, with heparinized syringes. Plasma was isolated after centrifugation at 800 *g* for 10 min. Plasma chol concentration was estimated using GT lab kit under manufacturer's instructions (CHOD/PAP, GT Lab). The initial plasma chol level and body weight were similar in both groups.

### Semen collection and handling

Ejaculated semen from both groups, HCR y NCR, was collected by an artificial vagina [18] from fertile New Zealand rabbits (6–15 month old), in accordance with the Guide for Care and Use of Laboratory Animals [19]. Two ejaculates were monthly obtained from each male, and then stored at 37°C until evaluation 15 min after collection. Samples containing urine and cell debris were discarded whereas gel plugs were removed. Semen samples were immediately assessed for physical parameters as aspect, color, volume and pH. Percentages of viability and morphological abnormalities were determined after a vital Eosin stain [20], (eosin 0.5% was prepared by diluting Y eosin in phosphate buffer saline, PBS: 200 ml were obtained diluting a Sigma tablet in pure water. Final concentration: 0.01 M phosphate buffer, 0.027 M KCl, 0.137 M NaCl, pH 7.4). Non stained cells were considered alive and expressed as percentage of total sperm cell counted in 40 µl of semen. Cell counting was performed on a slide mixing a drop of semen and eosin solution (semen drop plus eosin drop were placed between slide and cover slide) under 400 X magnification in a bright field microscope. This microscopy preparation was also used to evaluate sperm morphology [21]. In all cases 200 sperm cells were counted. After that, semen samples from both groups were diluted (1∶50, v/v) with warmed PBS and sperm motility of diluted samples was evaluated at 250 X under a phase-contrast microscope maintained at 37°C. Motility (progressive and in situ) was expressed as percentage of motile sperm over 200 cells. At the same time, cell concentration of diluted samples was estimated using a Macler counting chamber (Sefi-Medical Instruments, Israel). Finally, samples were washed twice by centrifugation - resuspension at 600 *g* for 10 min in PBS to remove seminal plasma. The final pellet was resuspended with PBS (20 to 200 µl, depending on the pellet volume). Then, the sperm suspension was adjusted to 5–10×106 cells/ml with BWW medium [22] and incubated in 35 mm Petri dishes (Corning®) under conditions that support capacitation during 16 h (Capacitation conditions: BWW supplemented with 4 or 40 mg/ml Bovine Serum Albumin (BSA fraction V), 20 mM NaHCO3, 37°C, 5% CO2, 95% air, time: 16 h. Four mg/ml was chosen using an average of the concentrations previously used by the group of Visconti [11], [23] and 40 mg/ml BSA was used following Guidobaldi's paper [24] in attempt to capacitate HCR sperm, since the first concentration failed to trigger the classical phosphotyrosine (p-Y) pathway. Sperm capacitation was determined by p-Y proteins and the proportion of AR induced by progesterone.

### Membrane cholesterol detection

Sperm cells (capacitated or not) were fixed with 4% paraformaldehyde in PBS 30 min at room temperature (RT) from both rabbit groups. Samples were then washed three times with PBS and centrifuged 15 min at 800 *g*. Sperm pellets were incubated with 0.15 mM (final concentration) of filipin complex 60 min in PBS (protected from light). A stock solution of 7.6 mM filipin was made by dissolving the filipin complex or filipin III in dimethylsulfoxide (DMSO) and stored frozen preserved under a nitrogen atmosphere inside microtubes. Then cells were washed once with PBS and centrifuged 15 min at 800 *g*, Cells were mounted with PBS-glycerol (50% v/v) for fluorescence microscopy analysis (380 nm Ex and 475 Em, Nikon). Sperm fluorescence was observed and recorded with a Hamamatsu C4742-95 camera attached to inverted microscope NIKON TE-2000. Sperm head fluorescence intensity was estimated by Image J software (n = 30) (Analyze option, histogram function applied to a delimited area  =  sperm head or acrosome area; *ImageJ 1.32j, http://rsb.info.nih.gov./ij/Java1.3.1_03*). Preliminary results indicated that filipin signal was preferentially distributed over the acrosomal region. Therefore, this condition was normalized as the ratio between acrosome and total head fluorescence: I =  Ai/Hi (I: index, Ai: fluorescence intensity corresponding to the acrosome region, Hi: fluorescence intensity corresponding to total sperm head surface). This index was relative and named as relative fluorescence index (RFI). RFI was calculated for all conditions and established for each sperm cell. Then, the percentage of cells with RFI ±1 was plotted for HCR and NCR.

### Cholesterol analysis

Total lipids from centrifuged spermatozoa were extracted following the instructions indicated by Laboratorio de Servicios y Ensayos (I.N.T.I.-Frutas y Hortalizas, Luján de Cuyo, Mendoza, Argentina). Cholesterol concentration was determined by Gas Chromatography (AutoSystem XL, Perkin Elmer) and is reported with respect to 10^9^ spermatozoa.

### Membrane integrity

PM integrity was evaluated by Hypo-Osmotic Test (HOS-T), [25]. Sperm cells were incubated in a hypo-osmotic solution (25 mM sodium citrate, 75 mM fructose in water) for 30 min at 37°C and then evaluated under phase contrast optic microscopy. Swelling of sperm cells was identified as changes in the shape of the tail. At least 100 cells were counted, and the proportion of spermatozoa that showed swelling in the hypo-osmotic solution corresponds to (not damaged) spermatozoa with membrane integrity and normal functional activity.

### Transmission electron microscopy

Samples were fixed 2 h at 0°–4°C adding to sperm suspensions (PBS) equal volume of fixative solution consisting of 4% paraformaldehide (w/v), 4% glutaraldehyde (w/v) and 20% piric acid (v/v) saturated in PBS [26]. Fixed sperm were washed then by centrifugation-suspension in fresh PBS for 10 min at 600 *g* (IEC, centrifuge). Then sperm cells were centrifuged –equal time and force– and sperm pellets were post-fixed adding 30 µl of 1% OsO4 (w/v) overnight at 4°C. Osmified samples were dehydrated in ethanol-acetone (up to absolute acetone) and embedded in epoxy resin (Epon 812, Pelco). Ultra-thin sections were obtained by Ultracut equipment (Leitz), stained with classical uranyl acetate and lead citrate TEM stain and examined with a Zeiss EM 900 (Zeiss, Oberkochen, Germany) at 80 kV.

### Phospho-tyrosine evaluation (sperm capacitation status)

Following an incubation period of 16 h, spermatozoa from HCR and NCR were concentrated by centrifugation 15 min at 850 *g* at RT, washed twice in 1 ml of PBS containing 0.2 mM Na3VO4 (unspecific phospahatase inhibitor) at RT and resuspended in sample buffer (25 mM Tris, 0.5% SDS and 5% glycerol, pH 6.8), [27], without mercaptoethanol boiling for 5 min. After centrifuging at 10,000 *g* for 15 min, the supernatant was removed and frozen until used. Five percent of 2-mercaptoethanol was added to defrosted samples (final concentration) and they were boiled for another 5 min and subjected to SDS-PAGE using 8–10% mini-gels according to Laemmli [27]. Protein extracts loaded per lane were equivalent to 5–10×10^6^ sperm. Each gel contained dual-prestained molecular weight standard (Bio Rad, Hercules, CA). Proteins were transferred to 0.45 µm nitrocellulose membranes (Bio Rad) and nonspecific reactivity was blocked by incubation over night with 3% Teleostean fish gelatin dissolved in washing buffer (TBS, Towbin's buffer plus 0.1% Tween 20). Blots were incubated with the anti-phophotyrosine antibody (clone PY20, ICN Biomedicals) 1∶5000 in blocking buffer for 1 h at RT. Biotin-conjugated anti-mouse IgG (Sigma) was used as secondary antibody (1∶1250) and horseradish peroxidase-conjugated extravidine (Sigma) was added at the end (1∶750), both in blocking buffer with a period of incubation of 1 h at RT each. Excess first and second antibodies were removed by washing three times for 10 min each in washing buffer. Detection was accomplished with an enhanced chemiluminescence system (ECL; Amersham Biosciences) and subsequent exposure to Blue Sensitive Cole-Parmer X-ray films (Cole-Parmer Instrument Company) for 5–30 s. In order to bypass membrane signal triggering PKA pathway, 1 mmol/L db-cAMP plus 100 µmol/L IBMX were added during the 16 h-capacitation period.

### Acrosome reaction (AR) assay

Capacitated sperm from HCR and NCR were incubated an additional 15 min with (induced reaction) or without (spontaneous reaction) progesterone in similar conditions (10 µM progesterone in DMSO), [28]. Another control was performed with an aliquot in the presence of DMSO in similar conditions to progesterone. AR was stopped and evaluated simultaneously by Triple Stain technique [29]. At least 300 cells were scored from each rabbit (in all conditions) to evaluate acrosomal reaction. For each experiment, AR percentage was calculated as percentage of reacted sperm over 300 sperm cells as: (*Number of reacted sperm induced by progesterone* - *Number of* spontaneously *reacted sperm*) *×100/300 =  AR percentage*. This percentage was first established for NCR and considered the control AR status. AR index was calculated as a percentage of this value (AR index). In this way AR index expresses the 100% for sperm from NCR and the percentage decreasing in HCR sperm.

### Statistical Analysis

Data were analyzed using statistical package software *GraphPad Prism 4* (http://www.graphpad.com/prism/Prism.htm, *San Diego*, *CA*, *USA*). Unless otherwise expressly noted, results in the text, table, and graphs are reported as means ± SEM of at least three independent experiments performed in duplicate. Differences between groups were evaluated by the Student's t-test considering a P value of less than 0.05 as statistically significant.

## Results

### Rabbit's cholesterolemia and body weight

**Figure 1:**
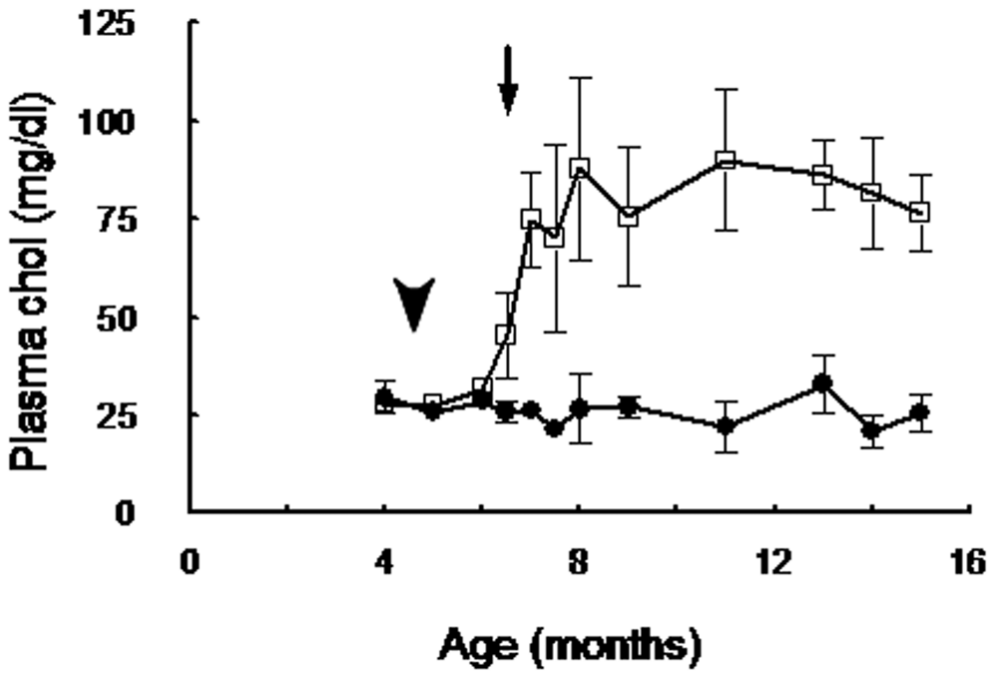
Rabbits fed with fat-rich diet increased plasma cholesterol. Plasma cholesterol concentration from NCR (•) and HCR (**□**) during the 11 experimental months. Values are expressed as mean ± SEM. Arrowhead indicates fat intake start in HCR, and arrow indicates the moment from which HCR weight resulted significantly different from NCR, (p<0.001).

Feeding of male rabbits on a diet containing 0.05% cholesterol significantly (*p<*0.001) increased total cholesterol level in the serum (Figure 1) without showing significant difference in body weight throughout the experimental time (body weight at the end of the experiment: 4.08±0.17 kg NCR and 4.37±0.24 kg HCR; data not shown), and body mass index (BMI) did not present difference between groups (and 17.65±1.36 NCR and 17.38±1.59 HCR, estimated at the end of the experiment, data not shown).

NCR displayed a low constant cholesterolemia (26.10±3.40 mg/dl), compared to the literature [30] throughout the study, whereas in HCR it was significantly increased (45.56±11 mg/dl, Figure 1B) 45 days after they began to feed the ED (6.5 months of age; Figure 1B, arrow). Chol in HCR reached the maximum level (87.77±23mg/dl) at 3 months of ED.

### Semen quality parameters

**Figure 2:**
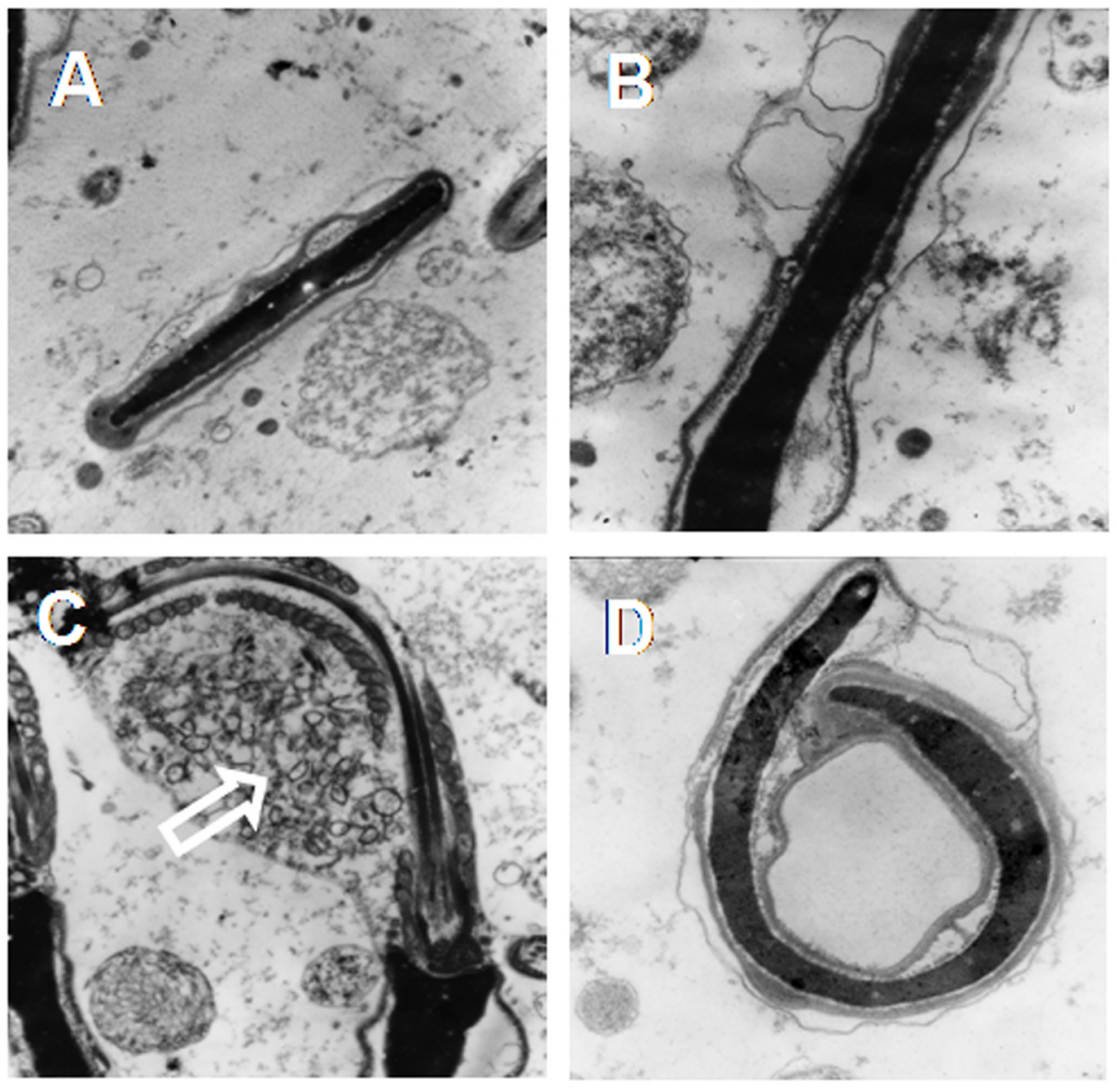
Ultrastructural changes. Transmission**-**electron micrographs of rabbit sperm heads from NCR (A) and HCR (B to D). Notice the small vesicles in the acrosome region in B and the long side fold of sperm head in D. Some sperm cells show the remaining residual body (white empty arrow, C). A and C, X 12,000; B and D, X 20,000.

The semen characteristics of NCR and HCR are summarized in Table 1. Semen pH, sperm concentration and vitality were not affected by dietary cholesterol. However, ejaculate volume and sperm motility significantly decreased in HCR. Moreover, sperm from HCR showed increased number of morphological alterations compared to NCR. Among other changes, there were two clearly noticeable: atypical sperm heads with the appearance of “folded head” -head fold along the major axe-, and the presence of cytoplasmic droplet. Ultrastructure of control (NCR) sperm head (Figure 2A) contrasted with electron-lucent membrane vesicles inside the acrosome (Figure 2B), remaining tail drop (Figure 2C) and sperm head folded along the major axe (Figure 2D). Nucleus, chromatin and mitochondrias showed normal morphological structure in both groups (data not shown).

**Table 1:** General characteristics of fresh rabbit semen samples (mean ± SEM).

	NCR	HCR
Volume (µl)	759,8±68.66	432.2±45.6*
pH (mean ± SD)	7.5±0.5	7.5±0.25
Sperm viability after eosin staining (%)	88.8±1.28	85.8±1.15
Sperm concentration (×10^6^/ml)	629.2±90.62	521±118.4
Total Motility (% A+B+C)	76.8±3.3	54.6±4.1*
Total sperm abnormalities (%)	21.1±2.4	33.6±3.5*

*p<0.001, *n* = 25.

### Sperm membrane cholesterol

**Figure 3:**
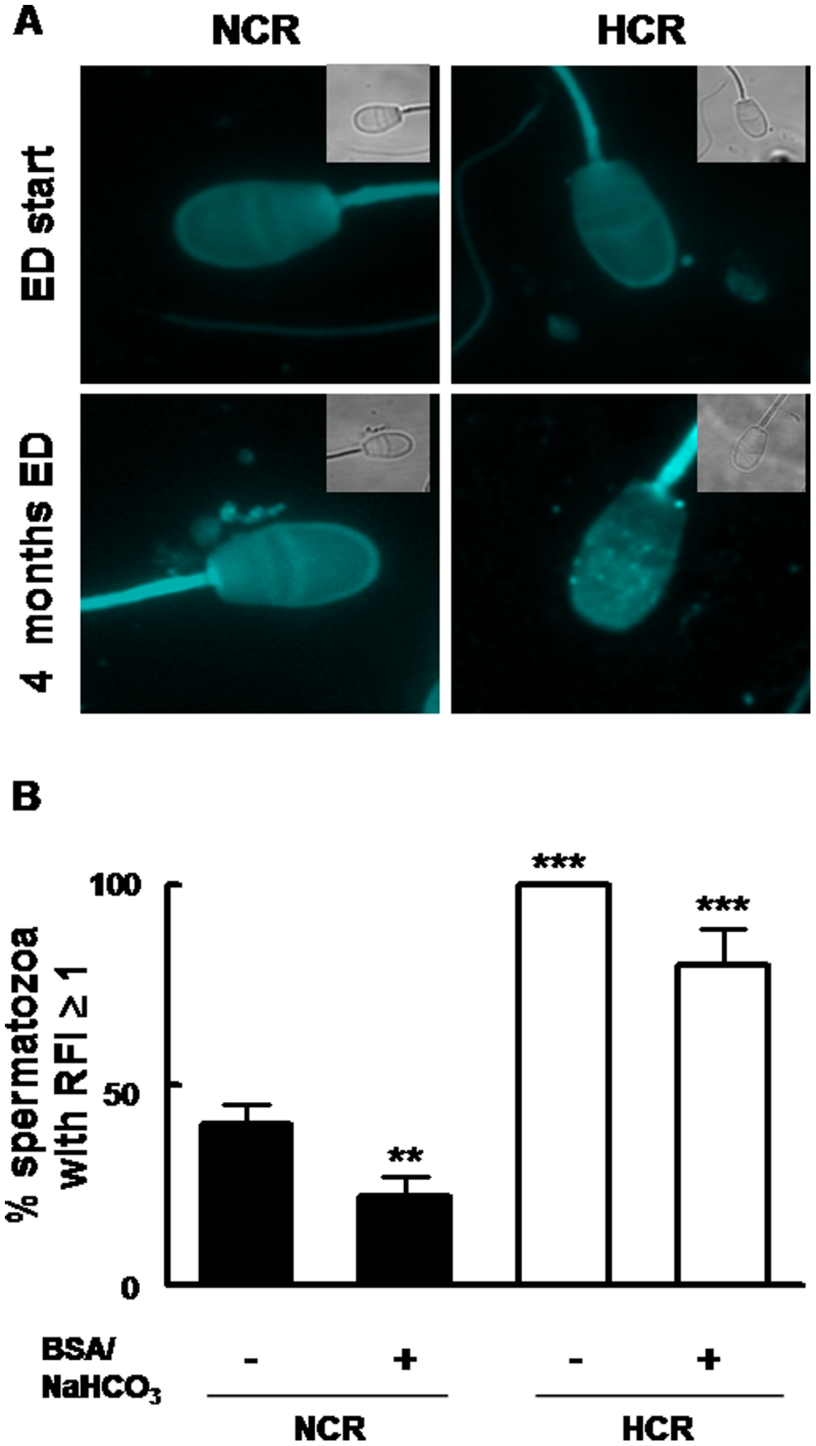
Sperm membrane cholesterol was elevated in HCR. A: Fluorescence micrographs showing cholesterol content in plasma membrane of ejaculated rabbit spermatozoa detected by filipin probe. Images correspond to phase contrast (inset) and filipin-stained sperm cells (X 600) from NCR and HCR at the beginning of the experiment (first row, ED start) and four months later (second row, 4 months ED). B: Bars represent RFI means (± SEM) in sperm cells isolated from NCR and HCR as described in “Materials and Methods”, from both conditions, non capacitated (- BSA/NaCOH_3_) and capacitated (+ BSA/NaCOH_3_). Asterisks  =  significantly different from control (**, p<0.01, ***, p<0.001).

Cholesterol dietary supplementation affected the cholesterol distribution in sperm cells after 4 months (Figure 3A). Percentage of cells with RFI ≥1 was signicantly higher for HCR sperms both in capacitation and non capacitation conditions (Figure 3B). At the end of the study, the cholesterol content of spermatozoa from HCR was 110 µg/10^9^ cells, whereas in the NCR was 64 µg/10^9^ cells.

### Sperm plasma membrane functionality

**Figure 4:**
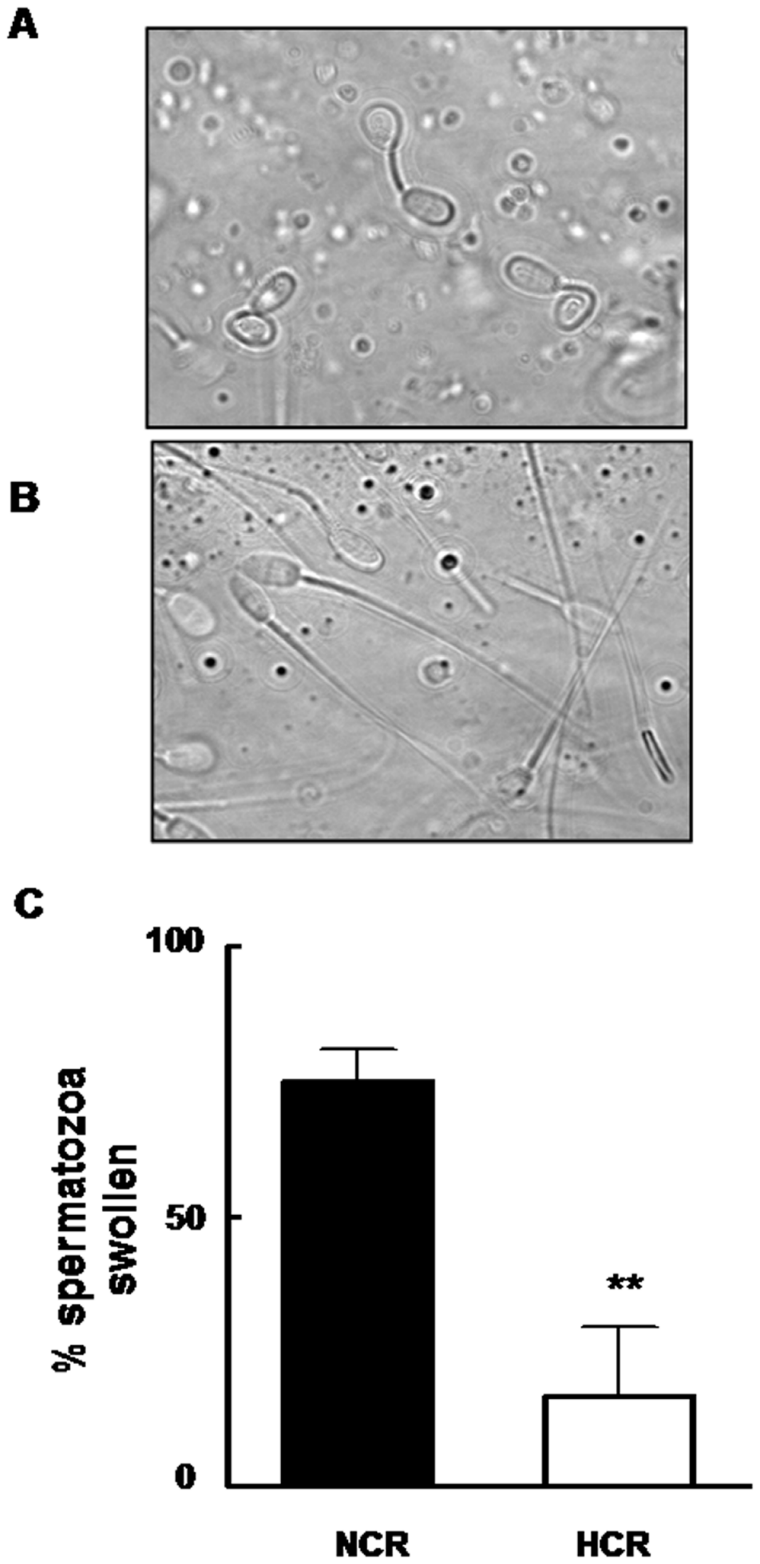
Saturated-fat consumption damaged sperm plasma membrane in rabbits. Photographs (X 250) represent sperm cell morphology after hypo-osmotic stress. A (NCR): normal coiled tails and B (HCR): straight tails. C: Bars represent the percentage (means ± SEM) of spermatozoa swollen from NCR (black bar) and HCR (white bar) rabbits. **  =  significantly different from control (p<0.01).

**Figure 5:**
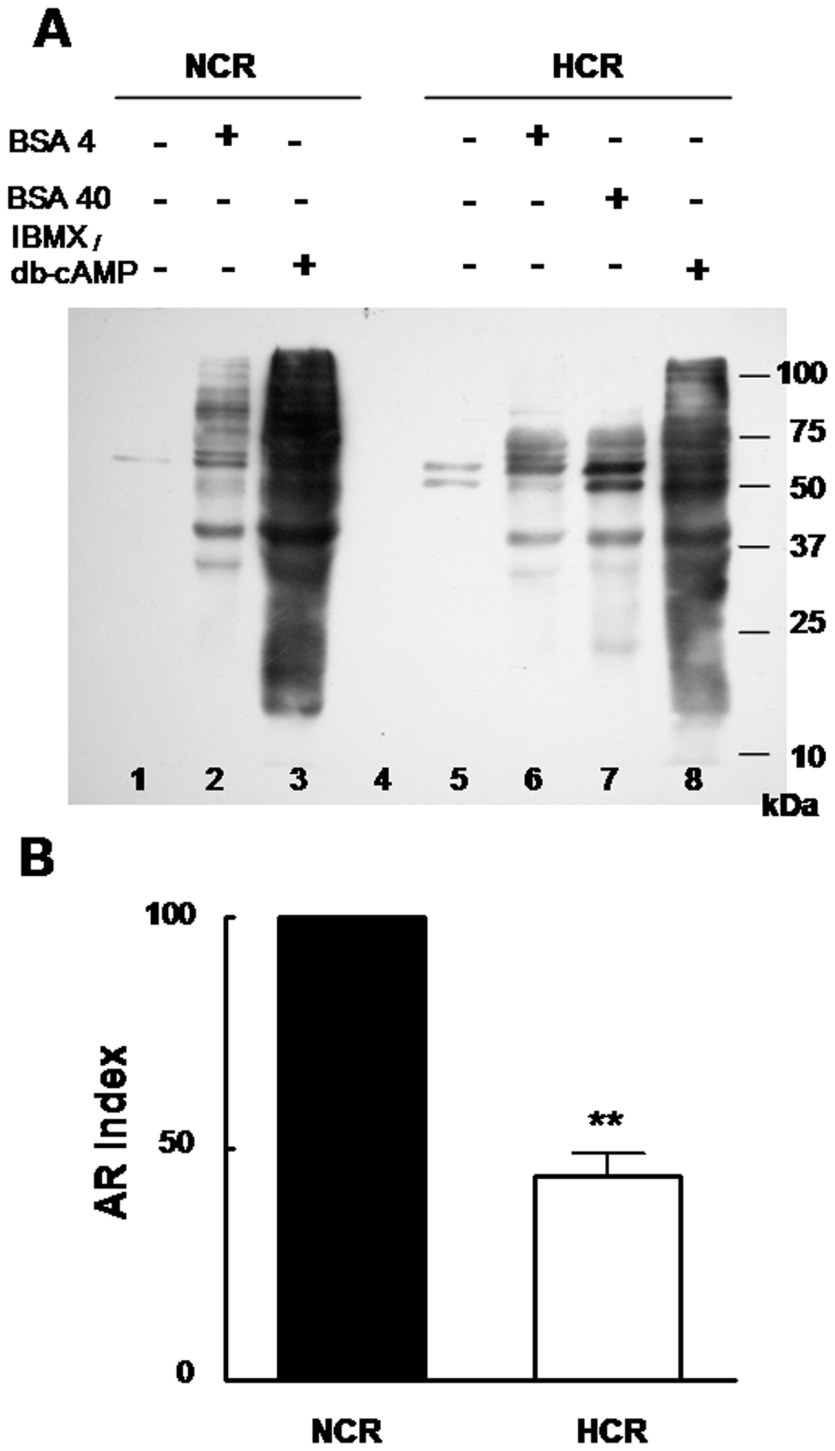
Hypercholesterolemia altered sperm capacitation and acrosome reaction. Protein tyrosine phosphorylation (A) and acrosomal exocytosis index (B) of spermatozoa from control (NCR) and HCR. Non capacitated: culture medium without (−) and capacitated with (+) BSA 4 (4 mg/ml), BSA 40 (40 mg/ml), IBMX (100 µmol/L)/db-cAMP (1 mmol/L). A: phospho-Y proteins showed different patterns ranging from one band (control-non capacitated, approximately 60 kDa) to many bands (capacitated with IBMX/db-cAMP, from over 20 to 100 kDa). Notice that the p-Y pattern differed between NCR and HCR: Non capacitated sperm presented one/two phosphorylated proteins (figure A, lane 1) but under capacitation conditions NCR showed wider molecular weight range of p-Y proteins compared with HCR (lanes 2 and 6). The experiment was performed at least three times and representative blot is shown. B: Bars represent AR index after 10 µM progesterone sperm incubation. AR index corresponds to normalized data (see Materials and Methods). ***  =  significantly different from control (p<0.001).

Spermatozoa from HCR showed a reduced sperm membrane response to the hypo-osmotic swelling test (Figure 4A and B) and to the induction of protein tyrosine phosphorylation under capacitation conditions (Figure 5A lane 6 and 2). Spermatozoa from HCR did not show the same pattern of p-Y bands compared to control either under non capacitating (Figure 5A, lanes 1 and 5) or capacitating (Figure 5A, lanes 2 and 6) media. The addition of tenfold amount of BSA to capacitation medium slightly improved the p-Y signal (Figure 5A, lanes 6 and 7) but not comparable to NCR p-Y pattern (Figure 5A, lanes 7 and 2). The treatment with two PK-A pathway stimulating compounds, db-cAMP plus IBMX, restored protein p-Y when chol removal signal was bypassed (Figure 5A, lanes 3 and 8). As expected, the increase in cholesterol concentration at plasma membrane level reduced the rate of acrosome reactive spermatozoa (Figure 5B).

## Discussion

Fat increment (0.05% chol) in standard diet promoted changes on sperm membrane chol concentration and distribution that ultimately altered membrane-coupled sperm specific functions: AR index decreased and p-Y pathway (capacitation) downgraded in White New Zealand rabbits. These changes were also associated with a reduction in motility and increase in sperm morphology alterations.

Plasma chol level reported for rabbit ranges from 35–53 mg/dl according to Harkness and Wagner [30]. However, we found that cholesterolemia from rabbits under control conditions maintained below that range all over the experimental period. On the other hand, it is widely known that saturated fat-enriched diets induce hypercholesterolemia in adult male rabbits [13], [14]. Accordingly, serum cholesterol level in HCR significantly increased at 45 days of ED diet.

The measures determined for different semen parameters (Table 1) were in agreement with standard estimations [31], [32], [33].

It is well known that diet lipids have consequences on sperm lipid composition [13], [14], [15], [34], [35]. Animals treated with flaxseed [34] or α-linolenic acid [35] improves semen quality by modifying sperm lipid composition. On the other hand, feeding saturated fat rich diets had been shown to trigger detrimental effects over rabbit semen [13], [14], [15]. In our results, ED diet did not affect semen parameters as pH and sperm viability. In previous results [15], high serum chol was associated with a decrease in sperm concentration and sperm motility. Our results confirm the reduction in sperm motility, although our experimental conditions differed in feeding time (longer) and fat intake (lower). In contrast to previous work [15], we found that semen volume significantly decreased in HCR. Probably, the feeding time used in the aforementioned study was not enough to affect the glands involved in seminal production and thus reduce the semen volume.

In our study, ED diet altered the filipin-sterol complexes distribution in the PM of the acrosomal region, indicating that hypercholesterolemia induces changes in sperm membrane lipid domains. This is in accordance to previous works [13], [14] though using a different methodology to detect membrane chol. The cholesterol concentration of spermatozoa we report is not in agreement with those of Castellini et al [6], but it has to be taken into consideration that in our work the samples were only centrifuged for spermatozoa separation.

Male infertility is correlated with sperm structure [36]. The morphological abnormalities we found can also be a sign of some degree of subfertility and could be further characterized. It would also be interesting to analyze if in a severe case of hypercholesterolemia those abnormalities correlate with male infertility. The unusual morphological abnormality we found (long-side folding of sperm heads) had not been reported and the mechanism underneath became difficult to explain.

The higher level of cholesterol content in sperms from HCR was associated with a decrease in membrane fluidity. Chol incorporation to the lipid bilayer could have affected normal sperm membrane integrity [37]. In human sperm cells increased membrane chol (poor-quality sperm) and decreased membrane fluidity are connected events [38]. Results presented here and those from literature could explain the reduction in HOS-t response.

Sperm capacitation and progesterone-induced AR resulted seriously affected in animals under fat-rich diets, suggesting that the main defect might reside on the PM. Spermatozoa from HCR were unable to achieve normal levels of protein p-Y even with ten fold increase of BSA. Therefore, it is probable that sperm from HCR may have more than the chol-related deficiency, which impairs their ability to successfully capacitate. An association between high chol content and capacitation deficiencies in human spermatozoa has recently been observed [38]. Thus, defects in membrane dynamics and sperm functional quality are strongly associated events.

In the presence of PK-A pathway activators (db-cAMP + IBMX) sperm from HCR achieved similar pattern of p-Y proteins as NCR. Those compounds bypassed the sperm PM signal and directly acted on intracellular molecules, thus the main kinase systems involved in capacitation-associated sperm protein p-Y were not affected. The defect, therefore, should be upstream from the kinases.

We wondered whether changes in membrane chol would affect AR, as it is known that a decrease in its content favors whereas an increase inhibits AR [39]. Spermatozoa from HCR have diminished sperm capacity to AR under a well known stimulus. This is consistent with previous report [13] showing that chol-enriched diet leads to modifications in sperm AR kinetics.

In conclusion, increased fat in a nutritionally complete diet altered the chol content and distribution of rabbit sperm PM. The dietary high fat level had serious consequences on sperm specific functions that depend on membrane integrity/dynamics. At least, two of the proposed pathways involved in regulating sperm capacitation and AR via chol resulted compromised. This outcome could resemble high-fat men consumers, with critical consequences on semen quality and therein on male fertility. Such study has also a clinical concern as hypercholesterolemia is a health global social problem [40]. Cellular events implicated will have to be understood to make harder progress not only in diagnosis and treatment but also in prevention.
